# Report of a Case of Apparent Disease of Antrum of Highmore

**Published:** 1899-04

**Authors:** W. H. Morgan

**Affiliations:** Nashville, Tenn.


ARTICLE IV.

### REPORT OF A CASE OF APPARENT DISEASE OF ANTRUM OF HIGHMORE.

DR. W. H. MORGAN, NASHVILLE, TENN.

Read before the Tennessee State Dental Association, July 5th, 1898.

About the second week in April, 1897, according to the patient's best recollection, she had extracted the first upper left molar, which had been giving her some trouble. The extraction, however, not relieving the sufferer, her dentist cauterized the alveoli with nitrate of silver, and finally, about the first week of June, brought his patient to my father, who diagnosed the trouble emphyemia of the antrum. After making an opening and establishing thorough drainage, he treated it, using the ordinary remedies in such cases, and dismissed the case apparently relieved in about a week. Soon after her return home, notwithstanding her efforts to keep up drainage and antiseptic washing, she began to suffer again very severely, and went back to her dentist, who, about the last of August, made a second opening in the antrum. From this time until the 22nd day of January he continued to keep up drainage, using everything which had been suggested by leading writers, with very little apparent benefit.

On Sunday, the 23rd day of January, assisted by Drs. Crockett and her former dentist, I anesthetized the patient, extracted the second molar and with an electric engine made an opening into the antrum as large as my little finger, made a careful exploration of the cavity to discover the presence of any foreign body that might be there. The result of this examination was that we found nothing present. The patient was in a comparatively comfortable condition until the Wednesday following, when she was taken with the most violent attack of suffering, which pain ceased after the dressing of iodoform gauze had been changed. This attack recurring at about the same hour of the day, sometimes an hour later, from Wednesday until the following Sunday, when we were driven to the conclusion (notwithstanding the fact that the dressing seemed to give great relief) that it was neuralgia, and of a malarial origin. Dr. Crockett prescribed muriate of quinine, and in a few days the patient was apparently entirely relieved. The amount of discharge at this time had diminished until it was almost gone. About two weeks later the neuralgia began again, and at this time we traced it, we thought, to a third molar in a state of partial eruption. The extraction of this tooth gave temporary relief, and at the end of two weeks the discharge still continued more or less, at times almost disappearing.

I concluded that the best thing to do was to insert a drainage tube, through which it would be possible for the patient to keep the antrum thoroughly cleaned, yet exclude food and permit perfect drainage; that if such appliance could be constructed the patient would be more comfortable and would not necessitate her remaining in Nashville longer. This tube was made and inserted on the 31st day of March. After wearing it a week she was permitted to return to her home, Decatur, Ala., in better health and in a greater state of comfort than she had been for twelve months. On Saturday, the 9th, however, she began to suffer again very greatly, and the following night returned to Nashville, When the tube was removed I discovered that a fungus growth had grown over the end of the tube, cutting off all possible chance for drainage. After removing this and re-establishing drainage, relief was obtained. This, however, did not continue long, for the pain previously alluded to began to return at intervals, and quinine does not relieve it.

The patient is very much run down, has recently been suffering from a bilious attack, and is in no condition to undergo another operation, and as there is little hope that her physical condition may improve in this climate, we have decided that a change is best for her, and she goes to Buffalo, where her father resides. I have not recently made any great change in the treatment of the case, save keeping it open and the cavity thoroughly washed. Since it was opened on Sunday, in January, there has not been any disagreeable odor, which had before that time made life a burden to her.

The following is an account of what was done with her at the Manhattan Eye and Ear Hospital, Park Avenue, New York, furnished me through the kindness of the house surgeon: According to his own and Wyeth's opinion Berens removed a large piece of the anterior alveolor process sufficient to easily insert his finger into the cavity of the antrum. The lower part of the cavity seemed perfectly healthy, but in the region of the ethmoid cells a large amount of polypoid tissue was found. This was carefully curetted away. The middle turbinate was next attacked, and a large portion of it removed sufficient to make a free opening into the antrum. Through this the ethmoid were investigated and found to be diseased. They were broken down and curetted away. Every step was taken with the greatest care and caution, and every portion of the antrum was thoroughly explored. The operation consumed over two hours, so great was the care exercised. The cavity was very tightly packed with sterilized gauze, which was always allowed to remain till the following night, when it caused such severe pain that he removed it and repacked it tightly. The following day it was removed for good and the cavity left free. From that time on the cavity was drenched every hour with a saturated solution of boric acid very hot. A spray of menthol, 10 grains to the ounce of benzonal, was used freely, greatly lessening the pain. After a week, when the discharge was somewhat lessened, the cavity was kept freely dusted with a calendulated preparation of boric acid. The relief following its use was marked and the cessation of the discharge noticeable. From time to time there appeared in the cavity small masses of granulations, which were kept in check by applications of orthochlorphenol. As the discharge lessened the periods between the douchings became longer until finally the discharge ceased. For a week before she left Berens kept the cavity closed with a plug of chewing gum. This was allowed to remain undisturbed for two days at a time, and at each removal showed no evidence of discharge. The opening was then allowed to close. The severe pain following the operation lasted about a week and disappeared with the inflammation and swelling of the tissues around the wound. When she left her appetite was fair and she had gained in weight and her color was very good. Berens and Wyeth both think the cure is permanent; and it looks so to me.

FROM T. PASSMORE BERENS, NEW YORK.

Henry W. Morgan, M. D., D. D. S., 211 High Street, Nashville, Tenn.:

Dear Doctor.—At the request of Miss Bellinger and Dr. Hale, I send you a short account of what I found and what I did with Miss Bellinger's case. After extracting the canine tooth from the affected side, I sawed through the alveolar process from the side of the incisor to the posterior end of the process, then described a cure with the saw through the bone forming the outer wall of the antrum, reaching almost to the level of the malar bone, thus gaining a good view of the interior of the antrum. I found a depression filled with granulation tissues over the second incisor tooth. I found also in the dome of the antrum many polypi, some of these occluding the orifice into the nose. After removing these I enlarged the opening into the nose and entered the ethmoid cells, which I found to be much diseased, full of pus and granulation tissue.

It is an interesting question of etiology whether the nasal condition caused the antrum disease or vice versa. Miss Bellinger made an uninterrupted recovery and was discharged. A week after her discharge she was seized with pain and returned to me. I found a band of cicatricial tissue stretching from the outer wall of the nose to the nasal septum and including a portion of the middle turbinate body. Irritation of this cicatrix markedly increased her pain; its removal has given her complete relief. Unfortunately, these cicatrices are apt to return, so that she probably will have some pain during the summer. In the fall I expect there will be sufficient reduction in size by contraction to enable a very slight operation to give entire relief.

It has been my experience, covering many of these cases, to find in almost all of them some nasal complication. Of course this does not apply to those acute or subacute cases due to the necrosis in or about the tooth. The microscopic report showed the presence of some foreign body imbedded in the tissue removed from the alveolus.

My object in leaving the inner side of the alveolus was, of course, to make a foundation for the future application of a plate. The orifice in the antrum will eventually entirely close up.—*Dental Headlight.*

